# Challenges and Solutions in Implementing eSource Technology for Real-World Studies in China: Qualitative Study Among Different Stakeholders

**DOI:** 10.2196/48363

**Published:** 2023-08-10

**Authors:** Bin Wang, Junkai Lai, Xiwen Liao, Feifei Jin, Chen Yao

**Affiliations:** 1 Peking University Clinical Research Institute Peking University First Hospital Beijing China; 2 Institute of Automation Chinese Academy of Sciences Beijing China; 3 Hangzhou LionMed Medical Information Technology Co., Ltd Hangzhou China; 4 Trauma Medicine Center Peking University People's Hospital Beijing China; 5 Key Laboratory of Trauma Treatment and Neural Regeneration Peking University Ministry of Education Beijing China; 6 National Center for Trauma Medicine of China Beijing China; 7 Hainan Institute of Real World Data Qionghai China

**Keywords:** electronic medical record, electronic source, eSource, challenge, real-world data, interoperability

## Abstract

**Background:**

eSources consist of data that were initially documented in an electronic structure. Typically, an eSource encompasses the direct acquisition, compilation, and retention of electronic information (such as electronic health records [EHRs] or wearable devices), which serves to streamline clinical research. eSources have the potential to enhance the accuracy of data, promote patient safety, and minimize expenses associated with clinical trials. An opinion study published in September 2020 by TransCelerate outlined a road map for the future application of eSource technology and identified 5 key areas of challenges. The background of this study concerns the use of eSource technology in clinical research.

**Objective:**

The aim of this study was to present challenges and possible solutions for the implementation of eSource technology in real-world studies by summarizing team experiences and lessons learned from an eSource record (ESR) project.

**Methods:**

After initially developing a simple prototype of the ESR software that can be demonstrated systematically, the researchers conducted in-depth interviews and interacted with different stakeholders to obtain guidance and suggestions. The researchers selected 5 different roles for interviewees: regulatory authorities, pharmaceutical company representatives, hospital information department employees, medical system providers, and clinicians.

**Results:**

After screening all consultants, the researchers concluded that there were 25 representative consultants. The hospital information department needs to implement many demands from various stakeholders, which makes the existing EHR system unable to meet all the demands of eSources. The emergence of an ESR is intended to divert the burden of the hospital information department from the enormous functional requirements of the outdated EHR system. The entire research process emphasizes multidisciplinary and multibackground expert opinions and considers the complexity of the knowledge backgrounds of personnel involved in the chain of clinical source data collection, processing, quality control, and application in real-world scenarios. To increase the readability of the results, the researchers classified the main results in accordance with the paragraph titles in “Use of Electronic Health Record Data in Clinical Investigations,” a guide released by the US Food and Drug Administration.

**Conclusions:**

This study introduces the requirement dependencies of different stakeholders and the challenges and recommendations for designing ESR software when implementing eSource technology in China. Experiences based on ESR projects will provide new insights into the disciplines that advance the eSource research field. Future studies should engage patients directly to understand their experiences, concerns, and preferences regarding the implementation of eSource technology. Moreover, involving additional stakeholders, including community health care providers and social workers, will provide valuable insights into the challenges and potential solutions across various health care settings.

## Introduction

### Background

eSources consist of data that were initially documented in an electronic structure. Typically, an eSource encompasses the direct acquisition, compilation, and retention of electronic information (such as electronic health records [EHRs] or wearable devices), which serves to streamline clinical research [1]. eSources have the potential to enhance the accuracy of data, promote patient safety, and minimize the expenses associated with clinical trials. In a 2020 publication by TransCelerate, a study conducted on opinions outlined a road map for the future use of eSource technology [2]. The study also highlighted 5 significant areas of challenges, which include clinical trial design, protocol and data collection, automated data exchange, safety and privacy, new roles, regulatory coordination, and cooperation [2]. The process of accessing and correcting the source data in real time during data collection may experience delays owing to various obstacles. These obstacles include the limited interoperability between EHRs and electronic data capture (EDC) systems, the presence of unstructured data such as researcher notes or comments, and the requirement to manually transcribe and handle certain data that are specific to the research and not included in the EHR [2]. Limited development, implementation, and evaluation of electronic resource solutions specific to EHR have been observed, despite the presence of multiple US Food and Drug Administration guidelines [1,3] and guidelines from the European Medicines Agency [4,5]. Over the past decade, numerous eSource solutions have been created, assessed, and enhanced [6-14], aiming to achieve an optimal technology that can bypass EDC data input altogether. This advanced technology is designed to directly extract source data from the EHR and seamlessly transmit them to an electronic case report form. Significant advancements in eSource research have been observed in various substantial initiatives, including the OneSource project [15], Electronic Health Records for Clinical Research [16], European FP7 Translational Research and Patient Safety in Europe (TRANSFoRm) [17,18], Electronic Health Records to Electronic Data Capture [19-21], and the Vulcan Health Level Seven International Fast Healthcare Interoperability Resources Accelerator [22-24].

Real-world data (RWD) refer to information regarding the health status of patients and the provision of health care, which is routinely gathered from diverse sources [25]. A real-world study (RWS) entails the collection of RWD within an actual environment, analyzing them to obtain real-world evidence regarding the practical value and potential advantages or risks associated with medical products [26].

### This Study

In 2019, the China National Medical Products Administration designated the Hainan Boao Lecheng Medical Tourism Pilot Zone as a testing ground for the RWS [27]. Subsequently, in December 2020, the Hainan Real World Research Institute initiated an eSource record (ESR) project [28] aimed at creating a comprehensive solution and tool for the collection, governance, and management of RWD within hospitals. In October 2021, the ESR project was officially released in China, and it was trialed in medical institutions in Boao Lecheng to support the implementation of the RWS [29]. For the design of the ESR system, refer to their recent proposal article [30]. In a real-world ophthalmology study, the researchers described the scenarios of ESR in hospitals, data standard transformation, and application effect assessment [28]. Retrospective and prospective studies can be supported by eSource or observational research data collection. The ESR has completed software deployment in nearly 10 medical institutions in Boao Lecheng. Researchers are working to pilot and promote the software in several large tertiary first-class hospitals in Beijing, China.

The background of this study concerns the use of eSource technology in clinical research. The purpose of this study was to examine the challenges and possible solutions regarding the implementation of eSource technology in RWSs by summarizing teams’ experiences and lessons learned from the ESR project in China. Readers interested in this study will include stakeholders who want to realize electronic transmissions from EHR to EDC systems, such as big data companies, medical system providers, and researchers in the field of clinical research. Similar to TransCelerate’s eSource initiative in the world, the researchers want to share the experience of ESR in China to promote global digital reform in the field of clinical research.

## Methods

### Collecting Design Recommendations for ESR Software

Qualitative research allows us to understand the participants’ experiences. After initially developing a simple prototype of the ESR software that could be demonstrated systematically, the researchers conducted in-depth interviews and interacted with different stakeholders to obtain relevant guidance and experience suggestions. The researchers selected 5 different roles for interview subjects: regulatory authorities, pharmaceutical company representatives, hospital information department employees, medical system providers, and clinicians. The sample size estimation was not applicable; the participants were stakeholders that the researchers could invite.

### Ethics Approval

This study was conducted in accordance with the principles of the Declaration of Helsinki. Ethics approval was obtained from the Peking University Institutional Review Board (number IRB00001052–21081).

### Informed Consent

Verbal informed consent was obtained from all the participants.

### Interview Guide

In-depth interviews with the interviewees were conducted using a flexible topic guide to ensure that the primary issues were covered across all interviews but enabled participants to introduce unanticipated issues. The interview topics were divided into 4 aspects: the view of eSource technology, the functional requirements of eSource technology in relevant fields, and the challenges and possible suggestions when applying ESR software to implement eSource technology.

Interview questions were designed according to the different roles of the interviewees (Textbox 1).

**Interview questions.**

**Introduction, background, and verbal informed consent:**

- Introduce the purpose of the interview process.
- Discussion of how interviews will be recorded, issues of confidentiality, anonymization, and informed consent.
- Verbal informed consent:
  - You agree to conversation being audio recorded?
  - You understand that the researchers will keep a written record of the interview but without anything that could identify you for future research?
- Introduce the concept of eSource and demonstrate the eSource record (ESR) system.

**Regulatory authorities:**

- What is your opinion on the use of eSource technology and the ESR system in regulation?
- What are your recommendations for regulating the use of real-world data (RWD) in clinical research?
- What features can facilitate the reduction of regulatory approval burdens?
- What functions does ESR need to support regulatory authorities? What are the barriers and recommendations?

**Pharmaceutical companies:**

- What is your opinion on the use of eSource technology and ESR systems in research?
- What are some suggestions for improving data quality when capturing research source data?
- What are the functional recommendations for facilitating the verification of source data?
- What functions does ESR need to support research? What are the barriers and recommendations?

**Hospital information department or medical system provider:**

- What is your opinion on the use of eSource technology and ESR systems in the medical system?
- What are the functional recommendations for transferring source data to other systems?
- What are the functional recommendations for facilitating the management of the source data required for study?
- What functions does ESR need to support the medical system? What are the barriers and recommendations?

**Clinicians:**

- What is your opinion on the use of eSource technology and ESR systems in the clinical environment?
- What tools are suggested to improve usability and efficiency for recording research source data?
- What are the functional recommendations for collecting research data while interacting with patients?
- What functions does ESR need to support the clinical environment? What are the barriers and recommendations?

### Data Collection

The researchers convened many web-based and offline meetings to summarize and sort out the challenges in and suggestions for advancing ESR software development by demonstrating ESR software prototypes. Many small expert interviews were conducted at academic conferences during the stage of designing and refining the theoretical framework of ESR software. During the formal development phase of the ESR software, there were discussions among various research teams, including big data companies, EDC system manufacturers, contract research organization teams, and medical system experts. These discussions took place in weekly small group sessions as well as in larger meetings. The interviews were recorded using a digital voice recorder. Audio recordings were transcribed and anonymized to protect confidentiality.

### Analysis

Interview transcripts were imported into NVivo (version 12; QSR International) qualitative data analysis software. The iterative process continued with data collection, coding, and analysis, followed by further data collection and analysis until saturation was reached, which occurred when the last few interviews fit the existing patterns and did not generate new ideas. Thematic analysis [31], using a data-driven inductive approach, was used to identify and analyze patterns and themes of particular salience for participants and across the data set using constant comparison techniques [32]. Two researchers (BW and FJ) designed a structured coding tree that began with inductive open coding. Once the core categories emerged, deductive selective coding was performed. Open coding was performed independently by the 2 researchers, and the derived core categories were compared in multiple rounds of discussions until all members agreed.

## Results

### Participant Information

After screening all consultants, the researchers identified 25 representative main consultants. These experts provided important advice. For specific personnel information, refer to Table 1. All participants, except for the clinicians, responded to the topic of using eSource technology in clinical research. Clinicians only interact with the hospital information system for patients’ daily diagnosis and treatment during their work; thus, they lack knowledge about eSource technology.

**Table 1:** List of participants.

Expert classification (participants, n)	Corporation
Regulatory authorities (7)	National Health Commission Health Development Research Center Data Center or National Drug and Health Technology Comprehensive Evaluation Center Policy Research Office, Information Center, NMPA^a^ China Center for Food and Drug International Exchange Hainan Drug and Medical Device Evaluation Service Center Hainan Boao Lecheng Medical and Drug Administration Hainan Institute of Real-World Data Hainan Institute of Real-World Data
Pharmaceutical companies (6)	Johnson & Johnson (Shanghai) Medical Equipment Company Limited Johnson & Johnson (China) Investment Company Limited Medtronic (Shanghai) Management Company Limited^b^ Poco International Medical Trading (Shanghai) Company Limited Poco International Medical Trading (Shanghai) Company Limited
Hospital information department (2)	Hospital information department
Clinicians (4)	Department of Medical Cosmetology International Optometry Center Department of Plastic Surgery Department of Hematology
Medical system provider (2)	Haitai Medical Information System Company Limited.
Contract research organization (2)	Hangzhou Tigermed Pharmaceutical Technology Company Limited Tigermed-Jyton
Big data company (2)	Jiaxing Yidisi Computer Technology Company Limited New Vision Medical Technology (Hangzhou) Company Limited

^a^NMPA: National Medical Products Administration.

^b^This entry refers to 2 individuals from the same company, specifically personnel #1 and #2.

### Functional Requirements of Different Stakeholders

**Figure 1:**
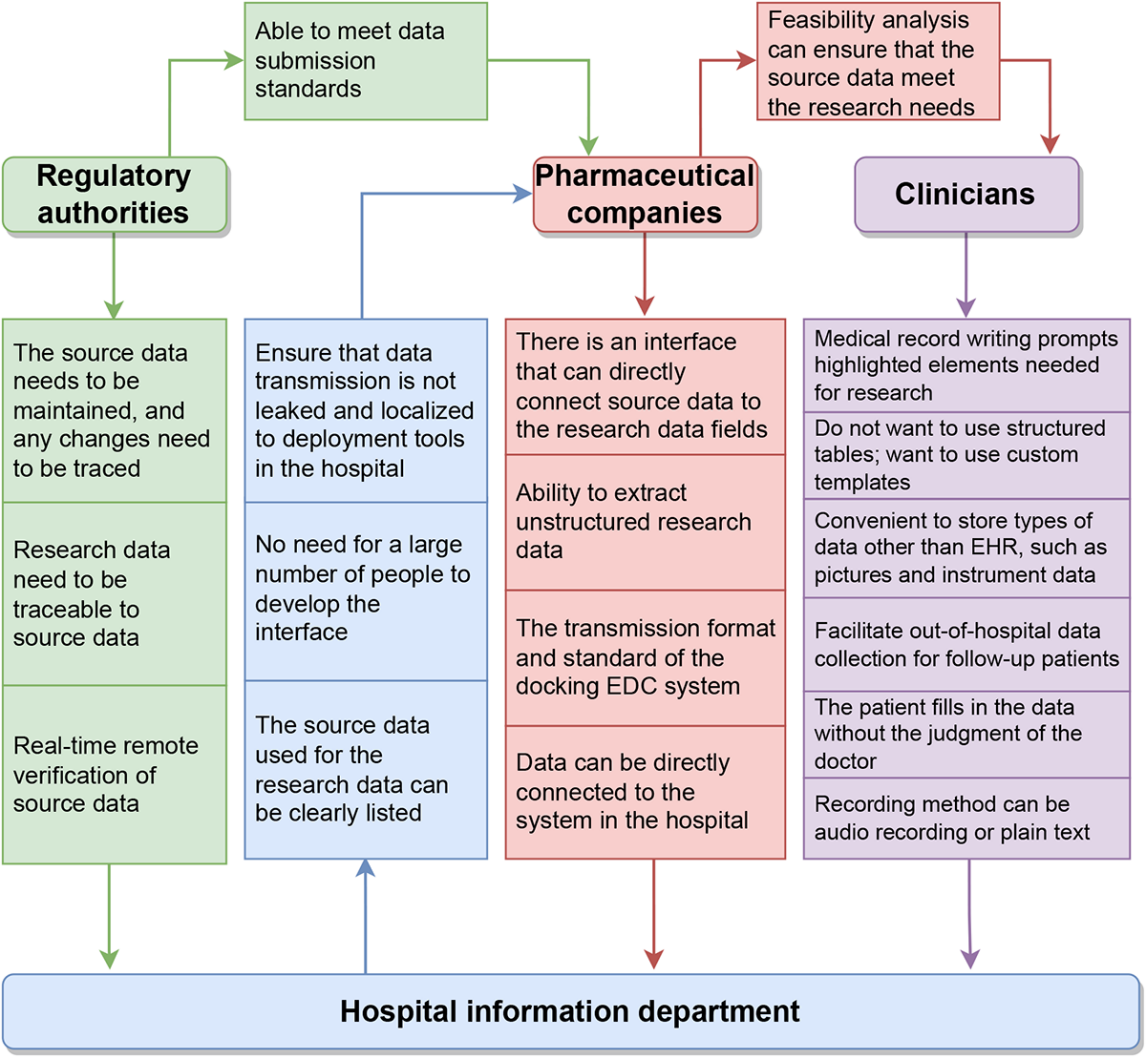
A summary of the proposed needs of different stakeholders. The arrow represents the direction of functional requirements from the proposer to the recipient. EDC: electronic data capture; EHR: electronic health record.

Different stakeholders have described the important functional requirements for implementing eSource technology (Figure 1). For each requirement, the proposer and the recipient of the requirement are shown in the direction of the arrow. For example, the regulatory authorities require the hospital information department to “keep the source data well maintained and leave notes about any changes,” and they require pharmaceutical companies to “comply with data submission standards” when submitting data. The hospital information department requires pharmaceutical companies to “clearly list the source data used for research”; that is, when hospital companies conduct clinical research, they must notify the hospital information department of the data they need for research rather than provide the full amount of medical data to pharmaceutical companies. The purpose of this is to reduce the workload of the hospital information department when extracting data, while ensuring the security of medical data and reducing the risk of data leakage. The clinician is the recorder of the research source data. The clinician’s focus is on the usability of the system. Therefore, it is assumed that even if the software meets the needs of other stakeholders, it will be rejected if it does not conform to the habits of clinicians. When clinicians are faced with a heavy demand burden without the right software for them, it is impossible for clinicians to record high-quality source data for research.

### Challenges and Recommendations for Designing the ESR Software

We compiled challenges and possible solutions for designing ESR software from various stakeholders. These details can be found in Tables 2 to 5 and Multimedia Appendix 1. Importantly, among all stakeholder-proposed solutions, those proposed by clinicians and regulatory authorities were primarily determined by the technical staff involved in the discussions. On the other hand, the solutions proposed by hospital information departments, medical system providers, and pharmaceutical companies are primarily focused on their own interests. To increase the readability of the results, the researchers classified the key results in accordance with the paragraph titles in “Use of Electronic Health Record Data in Clinical Investigations,” a guide released by the United States Food and Drug Administration [3].

**Table 2:** Challenges and possible solutions identified from the perspective of the hospital information department.

Theme	Existing challenges	Possible solutions
The medical record form needs to be flexible and configurable	Clinical habits are the greatest obstacle to restricting the use of medical record forms. Currently, the different medical record form structures used by each department take a long time to run and are determined after integrating the modification needs of clinical and hospital management. Therefore, whether medical forms can be configured to maximize their appearance for use by the clinician in the electronic health record system and whether the clinician is willing to collect source data and write the medical record in the ESR are important factors that determine clinical acceptance. Only when the ESR is highly configurable is it easy to complete the configuration of the project after it is deployed to the hospital and finally make the interface to transmit medical record documents.	ESR^a^ needs to add a medical record form configuration function and provide multiple types of question for users to flexibly design forms for different needs. ESR can consider the use of electronic paper technology to maximize the preservation of the form appearance similar to the form used by clinicians in the electronic health record system as well as the traceability and verification functions embedded in various electronic signatures.
Traceability and query associated with multiple comprehensive forms	When performing eCRF^b^ traceability, there may be a one-to-many situation between eCRF questions and forms of source data. When multiple forms are associated, the design of traceability logic may be complicated.	It is necessary to investigate the method and order of traceability checking by users. The design should conform to reading habits and realize automatic scrolling and positioning search functions as well as multiple form jumps and pop-up windows. It is best to decompose and recombine the eCRF questions and highlight the traceability content to facilitate traceability verification.

^a^ESR: eSource record.

^b^eCRF: electronic case report form.

**Table 3:** Challenges and possible solutions identified from the perspectives of the regulatory authorities.

Theme	Existing challenges	Possible solutions
Hospitals lack process management for real-world studies	Although the health department has detailed requirements for electronic health records, it does not meet the rigor of clinical trials. For real-world studies, hospitals lack process management that can be referenced to guide the construction of hospital informatization, and corresponding departments need to be established to manage data quality.	The design of the eSource record needs to consider the existing scientific research management model of the hospital and integration with the existing platform.
Comprehensive revision query	The eSource record has designed the function of revision trace tracking in each medical record form but lacks the function of revision tracking from the overall level of all enrolled patients, such as tracking how many patients have made a revision in a certain form.	From a regulatory perspective, a comprehensive revision query module can be provided. It can be modified by time, operator, form type, and different types of medical records and can locate, query, and jump to the records of the corresponding form. The interface of the medical record form needs to provide a traceability function for modifying records.

**Table 4:** Challenges and possible solutions identified from the perspectives of the clinician.

Theme	Existing challenges	Possible solutions
Availability of voice input	In Chinese hospitals, few physicians use voice input for various reasons. For many physician s with nonstandard pronunciation and accents, the effect of speech recognition will be poor. Because of personal habits, many physicians only use voice input to write medical records in the physician’s office without the presence of patients. However, its application scenarios are limited because of the need for a quiet and independent environment.	When the ESR^a^ chooses a speech recognition supplier, in addition to cost and after-sales issues, it is important to consider the recognition effect. To improve the effect of speech recognition, for specific projects, developers can consider collecting the user’s speech corpus to train the speech recognition model to obtain better results. For the use of voice input in the process of seeing a physician, it is necessary to judge and gradually adapt to the specific scenario.
Integration of ESR and EHR^b^ systems	The ESR should consider multiple docking options with EHR systems. Ideally, the ESR is integrated into the EHR system as a plug-in rather than as a standalone system, thus avoiding the need for clinicians to switch back and forth between different systems. If the ideal scenario cannot be achieved, it is necessary to ensure the synchronization of documents between the 2 systems to prevent clinicians from repeatedly recording EHRs. In addition, because the content of scientific research medical records is greater than that of medical records and given the complexity of the current physician-patient relationship, there may be a certain risk of medical disputes when the scientific research medical records recorded in the ESR are fully synchronized to the medical records of the EHR system. Therefore, it is necessary to specifically investigate the needs of clinicians and related departments of the hospital.	First, it is necessary to consider 2 options for the connection between the ESR and EHR, that is, whether the ESR supplementary source data needs to be written back to the EHR system. In addition, for the plug-in integration of the ESR in EHR, in-depth cooperation with medical system suppliers is required in the later stage. At this stage, the smoothness and fluency of the user’s switching process between the 2 systems should be ensured as much as possible to improve the user experience. For the synchronization scope of medical records and scientific research medical records, it is necessary to specifically investigate the needs of clinicians and relevant departments of the hospital.

^a^ESR: eSource record.

^b^EHR: electronic health record.

**Table 5:** Challenges and possible solutions from the perspectives of the pharmaceutical companies.

Theme	Existing challenges	Possible solutions
Compliance	Including whether there are any RWD^a^ standards that can be used, the differences between the standards for data collection and the existing CDISC^b^ standards, and how to interface challenges. Corresponding guidance documents are needed in the future so that application and promotion can be realized in terms of the management process, whether new technologies can meet regulatory requirements and the ESR^c^ uses NLP^d^ to extract research data formed by technology, how to conduct verification, whether it can be used as a data source for verification, and whether the regulatory authorities can accept this new method when conducting on-site inspections. Compliance also includes how to trace back when NLP extracts the wrong data because the patient answers incorrectly or the physician writes the wrong information and how these issues are communicated during the project review process by regulatory authorities.	Currently, there are many data standards. When designing ESR, it is necessary to consider the data standard transformation from source data to clinical research data. The clinical research data standard should choose the internationally recognized CDISC standard. For the source data, the corresponding data standard should be selected according to the data source. For example, it should be adapted to the specific data standard of HL 7^e^ used by the EHR^f^ system. To meet the regulatory verification requirements, the database integration and certification should be carried out in accordance with the requirements for the certified copy database in the regulations. For data traceability, if there is a source data error, a source error reporting reminder function needs to be designed. In ESR, the clinical research coordinator can report source error when checking the NLP extraction effect. For the EDC^g^ system, this error reporting function also needs to be added to send reminders to the ESR and EHR systems to remind clinicians to correct medical records.
Quantifying the value of the new approaches	Although current RWSs^h^ in China are different from traditional clinical trials in terms of design, data collection is still managed and implemented according to the requirements of clinical trials, which requires a great deal of human and economic costs.	In terms of cost saving, quality improvement, regulatory approval, and workforce saving, the corresponding indicators or recognized evaluation scales can be selected to evaluate the application effect of the ESR in the project.
Application of the ESR to retrospective data	In addition to being used for prospective data collection, the ESR can be used for retrospective data extraction. For retrospective data, currently, big data companies can undertake this type of business, but the fees are relatively high. When the ESR can actually be put into use, it can help the sponsor save on economic costs, or if the cost cannot be saved, the data quality can be improved. Reliance on a conventional big data company can cause compliance problems because the original data need to be transmitted from the hospital to the server of the company, and all data governance links cannot be effectively verified and traced. To solve the problem of data security, all data must be supervised by the hospital, and the ESR needs to complete the extraction of retrospective data within the hospital.	The ESR needs to be deployed locally in the hospital. For retrospective data extraction, it will meet the requirements of medical data security. Researchers can choose the completed RWD research project, use the ESR to extract retrospective data, and compare it with the previous manual data transcription method using EDC system to evaluate the quality of the ESR for retrospective data extraction, efficiency, and other indicators.

^a^RWD: real-world data.

^b^CDISC: Clinical Data Interchange Standards Consortium.

^c^ESR: eSource record.

^d^NLP: natural language processing.

^e^HL 7: Health Level Seven International.

^f^EHR: electronic health record.

^g^EDC: electronic data capture.

^h^RWS: real-world study.

### Interoperability and Integration of Systems

The challenges and recommendations provided by various stakeholders with regard to data standards, structured and unstructured data, and validation are presented in Textbox 2.

**Challenges and recommendations provided by various stakeholders.**

**Data standards**

- Compliance (proposed by pharmaceutical companies)
  - Challenges: Including whether there are any real-world data standards that can be used, the differences between the standards for data collection and the existing Clinical Data Interchange Standards Consortium standards, and how to interface challenges.
  - Recommendations: currently, there are many data standards. When designing an eSource record (ESR), it is necessary to consider the data standard transformation from source data to clinical research data. The clinical research data standard should choose the internationally recognized Clinical Data Interchange Standards Consortium standard. For source data, the corresponding data standard should be selected according to the data source. For example, it should be adapted to the specific data standard of Health level 7 International used by the electronic health record (EHR) system.

**Structured and unstructured data**

- Field mapping and data synchronization between the EHR and ESR systems (proposed by medical system providers)
  - Challenges: clinicians have performed a great deal of semistructured customization of personalized specialist medical records. Fields may not have standard naming, which increases the difficulty of mapping the fields between the 2 systems.
  - Recommendations: during transmission between the 2 systems, the semistructured fields in the EHR system should be converted into long text data and then synchronized to the ESR system for natural language processing extraction, thereby reducing the workload of fine-grained field mapping.
- Integration of ESR and EHR systems (proposed by the clinicians)
  - Challenges: the ESR should consider multiple docking options with EHR systems.
  - Recommendations: it is necessary to consider 2 options for the connection between the ESR and EHR; that is, whether the ESR supplementary source data needs to be written back to the EHR system. The smoothness and fluency of the user’s switching process between the 2 systems should be ensured as much as possible to improve the user experience.
- Timeliness (proposed by medical system providers)
  - Challenges: the introduction of any new system should first consider whether it can effectively improve the efficiency of clinicians and save time.
  - Recommendations: the ESR can improve the efficiency of clinicians in writing medical records by introducing technologies such as voice input and optical character or image recognition.
- Availability of voice input (proposed by the clinicians)
  - Challenges: for many physicians with nonstandard pronunciation and accents, the effect of speech recognition will be poor.
  - Recommendations: to improve the effectiveness of speech recognition, for specific projects, developers can consider collecting the user’s speech corpus to train the speech recognition model to obtain better results.

**Validation**

- Stability and reliability (proposed by medical system providers)
  - Challenges: stability and reliability require the ESR to avoid any downtime failures and bugs in system operation as much as possible to avoid affecting daily medical routines.
  - Recommendations: the ESR must undergo rigorous and extensive testing before it can be used clinically.
- Safety (proposed by medical system providers)
  - Challenges: the ESR must meet the requirements of medical data security, which is the first condition.
  - Recommendations: in-hospital localization deployment ensures that data do not leave the hospital and truly guarantees the security of medical data.
- Maintainability (proposed by medical system providers)
  - Challenges: maintainability is a requirement for the expansion and long-term use of the ESR.
  - Recommendations: in addition to daily maintenance, the ESR must be able to make corresponding adjustments and updates to its own products in a timely and flexible manner when any connected hospital information system products are updated and iterated or when hospital business needs change.
- Hospitals lack process management for real-world study (RWS; proposed by regulatory authorities)
  - Challenges: for RWS, hospitals lack process management that can be referenced to guide the construction of hospital informatization, and corresponding departments need to be established to manage data quality.
  - Recommendations: the design of the ESR needs to consider the existing scientific research management model of the hospital and its integration with the existing platform.
- Application of the ESR to retrospective data (proposed by pharmaceutical companies)
  - Challenges: for retrospective data, currently, big data companies can undertake this type of business, but their fees are relatively high. Reliance on a conventional big data company can cause compliance problems because the original data need to be transmitted from the hospital to the company’s server, and not all data governance links can be effectively verified and traced.
  - Recommendations: the ESR should be deployed locally in hospitals. Retrospective data extraction will meet the requirements of medical data security.
- Quantifying the value of the new approaches (proposed by pharmaceutical companies)
  - Challenges: although current RWS in China are different from traditional clinical trials in terms of design, data collection is still managed and implemented according to the requirements of clinical trials, which incurs a great deal of human and economic costs.
  - Recommendations: in terms of cost savings, quality improvement, regulatory approval, and workforce savings, the corresponding indicators or recognized evaluation scales can be selected to evaluate the application effect of the ESR in the project.

### eSource Principles for EHRs

The eSource principles for EHRs consist of 2 aspects: data originator and data modifications. These principles of EHR are presented in Textbox 3.

**eSource principles for electronic health record (EHR).**

**Data originator**

- Traceability and query associated with multiple comprehensive forms (proposed by the hospital information department)
  - Challenges: when performing an electronic case report form traceability, there may be a one-to-many situations between the electronic case report form questions and the forms of source data. When multiple forms are associated, the design of the traceability logic may be complicated.
  - Recommendations: it is necessary to investigate the method and order of traceability checks by users. The design should conform to reading habits and realize automatic scrolling and positioning search functions, as well as multiple form jumps and pop-up windows.

**Data modifications**

- Field mapping and data synchronization between the EHR and eSource record (ESR) systems (proposed by medical system providers)
  - Challenges: when the source data are modified, dealing with the data synchronization problem in various situations involves multiple links and personnel with different roles, and the logic is complicated.
  - Recommendations: the ESR should limit the number of revisions of medical records according to medical regulations, such as locking medical records after a certain period and not allowing further revisions. For the modified data synchronization problem, a variety of complex scenarios can be handled using a combination of web-based automatic real time synchronization and offline manual synchronization.

**Inspection, record keeping, and record retention requirements**

- Comprehensive revision query (proposed by regulatory authorities)
  - Challenges: The ESR lacks the function of revision tracking from the overall level of all enrolled patients, such as tracking how many patients have made a revision in a certain form.
  - Recommendations: from a regulatory perspective, a comprehensive revision query module can be provided. It can be modified by time, operator, form type, and different types of medical records, and can locate, query, and jump to the records of the corresponding form.

## Discussion

### Principal Findings

This paper introduces the demand dependencies, existing challenges, and possible suggestions of different stakeholders when implementing eSource technology in China. Figure 1 shows that the number of requirements that the hospital information department needs is large, which makes it impossible to realize all the requirements in the existing EHR system. This explains why the researchers developed standalone ESR software that acts as a bridge between the EHR and EDC systems. The emergence of ESR is intended to divert the burden of the hospital information department from the enormous functional requirements of the outdated EHR system.

The Vulcan Real World Data project [33] is developing methods to use the Vulcan Health Level Seven International Fast Health Care Interoperability Resources standard to retrieve relevant data from RWD sources, for now primarily EHR systems, and ultimately will transform that data into a format suitable for submission to pharmaceutical regulatory agencies. The solution of this project is currently limited to the use of EHR for retrospective data analysis. The challenge faced by the project is the semantic and syntactic differences in the data transmission process from the field of health care data to the field of research and regulation. A qualitative study [34] described the challenges of implementing electronic trial data collection in primary health care using TRANSFoRm. In this qualitative study, different stakeholders were interviewed, including general practitioners, practice managers, information technology leaders, and research staff. It was found that the installation process of TRANSFoRm software was time-consuming and difficult because of the complexity of the primary health care information system [34]. These complications are primarily because of the differences between the permissions and information system skills of different stakeholders [34]. Researchers have found difficulties in the application value of quantified eSource technology (Table 5). Nordo et al [35] proposed a set of standardized measures to evaluate human security, data quality, operational efficiency, and the cost of eSource solutions. The researchers can see that many of the core evaluation indicators proposed in this study are consistent with the content of the interviews.

The preservation of patient anonymity and the impact on the design of eSource technology systems is a critical aspect that warrants careful consideration. Maintaining the confidentiality of patient’s medical information is of utmost importance in the implementation of eSource technology. Anonymity plays a crucial role in establishing trust between patients and health care providers, ensuring that their privacy is respected and protected [36,37]. The preservation of patient anonymity has significant implications for the design of eSource systems. It necessitates the development of robust data security measures to safeguard patient information from unauthorized access and breaches. The design should include encryption protocols, user authentication mechanisms, and access controls to ensure that only authorized individuals have appropriate access to patient data. These measures are essential for maintaining patient confidentiality and instilling confidence in the use of eSource technology for RWS.

In addition, it is essential to acknowledge the potential involvement of other stakeholders in data collection beyond the 5 groups studied in this research. The process of treating patients extends beyond their time in the hospital, and eSource technology may need to capture data from their experiences in community health care and social care settings. Stakeholders, such as community health care providers, social workers, and caregivers, play important roles in these contexts [36,38]. Recognizing their involvement and understanding their perspectives is crucial for comprehensively addressing the challenges and potential solutions associated with eSource technology implementation in RWSs.

### Limitations

There are certain limitations in this study. First, this study focuses on summarizing important challenges and suggestions. Second, there are still differences between the content and organization of the study results and regulatory guidelines. Finally, the stakeholders the researchers have included are still limited, and future studies need to focus on more significant stakeholders, such as patients, community health care providers, and social workers, among others.

### Conclusions

This study introduces the requirement dependencies of different stakeholders and the challenges and recommendations for designing ESR software when implementing eSource technology in China. Experiences based on ESR projects will provide new insights into the disciplines that advance the eSource research field. Moving forward, the researchers acknowledge the need to expand the scope of this research to incorporate the patient perspective and involve a broader range of stakeholders. Future studies should engage patients directly to understand their experiences, concerns, and preferences regarding the implementation of eSource technology. By doing so, researchers can develop systems that prioritize patient privacy, uphold data security, and align with their needs and expectations. Moreover, involving additional stakeholders, including community health care providers and social workers, will provide valuable insights into the challenges and potential solutions across various health care settings.

### Summary of the Latest Experience of the ESR Project

**Figure 2:**
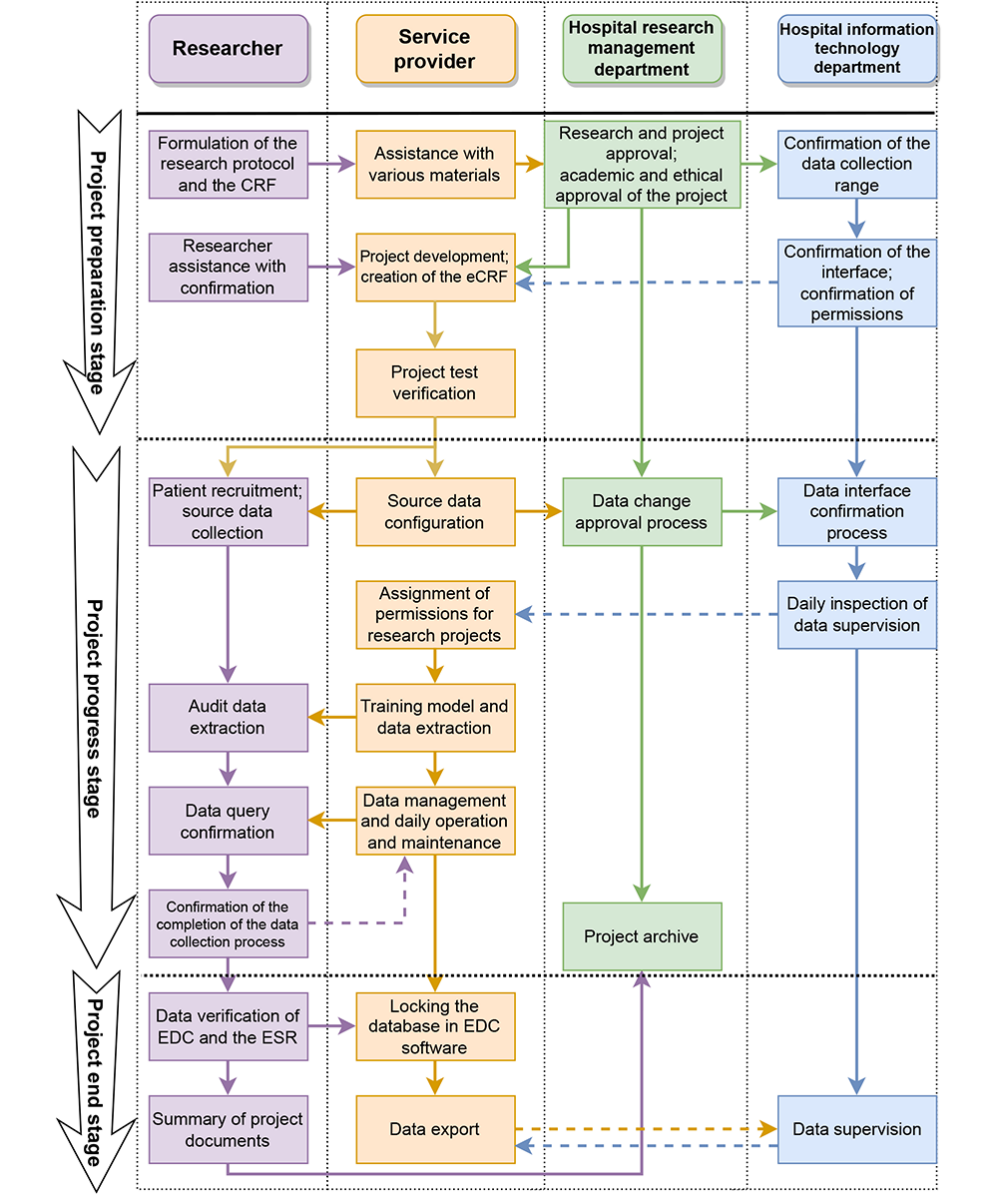
Management process of applying eSource record (ESR) tools in clinical research projects. CRF: case report form; eCRF: electronic case report form; EDC: electronic data capture.

It has been nearly 1 year since we conducted this qualitative study. As we continue to promote the trial use of ESR software in new research projects, the views delineated in this study may lag behind practical experience. Therefore, we have added recent progress results. We summarized the management process of applying ESR tools in clinical research projects (Figure 2). In this process, we differentiated the process based on the different stakeholders. The role of service providers corresponds to that of big data companies or medical system providers. At the same time, we summarized the standard operating procedures for the management responsibilities of different stakeholders (Multimedia Appendix 2). These new achievements will provide additional possibilities and implications for readers to understand the main content of the paper.

## Appendix

Multimedia Appendix 1 Challenges and possible solutions identified from the medical system.

Multimedia Appendix 2 Management responsibilities of different stakeholders.

## Abbreviations

EDC: electronic data capture
EHR: electronic health record
ESR: eSource record
RWD: real-world data
RWS: real-world study
TRANSFoRm: Translational Research and Patient Safety in Europe

## Data Availability

The data sets used and analyzed during this study are available from the corresponding author upon reasonable request.
