# Supplementary material for: Challenges and Solutions in Implementing eSource Technology for Real-World Studies in China: Qualitative Study Among Different Stakeholders
Source: JMIR Form Res. 2023 Aug 10;7:e48363. doi: 10.2196/48363 (PMC10450541; doi:10.2196/48363)
Supplement: Multimedia Appendix 1 [file formative_v7i1e48363_app1.docx]

**Table S1.** Challenges and possible solutions identified from the medical system provider’s perspective.

| Theme | Existing challenges | Possible solutions |
| --- | --- | --- |
| Timeliness | Timeliness means that after using the ESR^a^, clinicians cannot spend more time than they did using previous EHR^b^ systems. The introduction of any new system should first consider whether it can effectively improve the efficiency of clinicians and save time. | The ESR can improve the efficiency of clinicians in writing medical records by introducing technologies, such as voice input and optical character or image recognition. When evaluating the clinical application effect of ESR, it is necessary to incorporate the time spent into the evaluation index. |
| Stability and reliability | Stability and reliability require the ESR to avoid any downtime failures and bugs in system operation as much as possible to avoid affecting daily medical routines. | The ESR must undergo rigorous and extensive testing before it can be put into clinical use. |
| Safety | The ESR must meet the requirements of medical data security, which is the first condition. | In-hospital localization deployment realizes that data do not leave the hospital and truly guarantees the security of medical data. |
| Maintainability | Maintainability is a requirement for expansion and long-term use of the ESR. | In addition to daily maintenance, the ESR needs to be able to make corresponding adjustments and updates to its own products in a timely and flexible manner when any connected HIS^c^ products are updated and iterated or when hospital business needs changes. |
| Field mapping and data synchronization between the EHR and ESR systems | The EHR system contains many semistructured forms, especially for the needs of scientific research to collect data; clinicians have performed a great deal of semistructured customization of personalized specialist medical records. Fields may not have standard naming, which increases the difficulty of mapping fields between the 2 systems. | The ESR uses the most granular fields possible to synchronize semistructured fields with EHR. During transmission between the 2 systems, the semistructured fields in the EHR system are converted into long text data and then synchronized to the ESR system for natural language processing extraction, thereby reducing the workload of fine-grained field mapping. |
| Field mapping and data synchronization between the EHR and ESR systems | When the source data are modified, dealing with the data synchronization problem in various situations involves multiple links and personnel with different roles, and the logic is complicated. | The ESR should limit the number of revisions of medical records according to medical regulations, such as locking medical records after a certain period and not allowing further revisions. For the modified data synchronization problem, a variety of complex scenarios can be handled by a combination of web-based automatic real time synchronization and offline manual synchronization. |
| Field mapping and data synchronization between the EHR and ESR systems | There are certain challenges in how the ESR connects with past historical data to support retrospective research. | For hospitals with Clinical Data Repositories, choosing to connect only the data required for research will greatly reduce the workload of interface development. If the connection is difficult, the data can be manually exported and then manually stored in the certified copy database of the ESR. |
| Event linkage and reminders | How to use an event trigger mechanism to remind clinicians to make a doctor’s order for a certain inspection requirement in the eCRF^d^. | This part of the requirement is only possible when the ESR is part of the medical system provider’s HIS. |
| Event linkage and reminders | For adverse events in the eCRF, linking with separate systems in the hospital, such as the management of hospital feeling and the system for reporting critical values. | For the reminder function of adverse events, to connect with some independent systems of the hospital, it is necessary to re-evaluate the workload of interface development and the priority of such requirements. |
| Event linkage and reminders | The ESR can decompose the source data collection requirements in the eCRF, but how to use the business system of the HIS to notify clinicians in time will involve deeper intersystem connection issues, thus introducing a greater workload. | The connection between the ESR and HIS is not only about data transmission and synchronization but, more importantly, is about effectively linking the 2 systems and having a reminder function. Therefore, medical system suppliers need to use ESR technology and integrate this new technology into the existing medical system ecosystem to exert the application value of ESR. |
| Precise positioning and matching of clinical situations and visit points | The source data of the eCRF run through the entire diagnosis and treatment process, not just the writing process in the medical record. Visit points can be distinguished very accurately in clinical studies, whereas inpatients show up across visits. Therefore, the data sources of the eCRF forms of different visit points in the ESR may be scattered in diverse medical scenarios, which makes it impossible to accurately locate and match the corresponding visit points for clinical diagnosis and treatment. | The data collection point of the ESR needs to be able to flexibly add different time points according to the actual medical scenario. For data source matching between different visit points in the ESR and the eCRF form, the extensiveness of the data sources should be considered, and the eCRF form needs to be split and recombined to meet the data collection needs. |
| Precise positioning and matching of clinical situations and visit points | For some complex logical judgment processes, such as common inpatients having multiple laboratory test results that may need to be manually judged by researchers, the data synchronized from the laboratory information systems should be selected, such as which test results were obtained and which visits belonged to which patient. | When synchronizing laboratory information system data with ESR, it is currently possible only to manually mark and select the synchronized data. In the future, some filtering functions can be added according to actual scenarios, thereby reducing the time spent in this process. |
| Patients screened according to admission and discharge criteria | The eCRF entry and exclusion criteria were converted into logical conditions that can be used for screening in the EHR system, so it is difficult to screen suitable participants. | The formulation of logical screening conditions, in addition to relying on the function of the ESR, requires the EHR system to support the screening and retrieval functions of complex conditions. Therefore, when the ESR is actually implemented and connected to the EHR system, it is necessary to consider the product functions of the medical system supplier used by the hospital and then selectively develop the interface according to the actual situation. |
| Patients screened according to admission and discharge criteria | Some inclusion criteria may require researchers to manually judge based on patient medical history information rather than screen based on quantitative indicators, such as age and laboratory test indicators. Therefore, it is necessary to locate these inclusion criteria in free-text electronic medical record conditions to ultimately determine whether they will be selected. | Natural language processing technology can be used for searching and locating free text, so it can be considered to extract these logical judgment–based natural language processing and then manually make the final judgment of the ranking criteria, thereby reducing the time spent browsing medical records and improving work efficiency. |

^a^ESR: eSource record.

^b^EHR: electronic health record.

^c^HIS: hospital information system.

^d^eCRF: electronic case report form.
