# Supplementary material for: Challenges and Solutions in Implementing eSource Technology for Real-World Studies in China: Qualitative Study Among Different Stakeholders
Source: JMIR Form Res. 2023 Aug 10;7:e48363. doi: 10.2196/48363 (PMC10450541; doi:10.2196/48363)
Supplement: Multimedia Appendix 2 [file formative_v7i1e48363_app2.docx]

**Table S1.** Management responsibilities of different stakeholders.

| Role and responsibilities | | Description |
| --- | --- | --- |
| **Hospital information technology department** | | |
|  | **Interface** | |
|  |  | - Encapsulate and manage the data interfaces of various information systems in the hospital and establish an open management process for interface docking - Ensure that the interface management process is compatible and integrated with the good clinical practice process in the hospital |
|  | **Authority** | |
|  |  | - Interface authority management for managing the system, project, and personnel authorized by the interface - The approval of the authority needs to have corresponding rules and procedures, for example, when granting the authority of the ESR^a^ system based on the medical order data and which qualification certificates the ESR system manufacturer needs to provide |
|  | **Maintenance** | |
|  |  | - Handle interface data synchronization exceptions - When the manufacturer of the hospital information system is changed, the corresponding interface must be updated or added in a timely manner, and the ESR system party must be informed - Establish a mechanism to regularly check whether the interface works normally |
| **Service provider** | | |
|  | **Center operations** | |
|  |  | - System use training - Demand collection and feedback - Project docking and introduction - Promoting and publicizing implementation of the hospital’s project and the effect of system function services - Assisting contract signing, project approval, and other matters - Maintaining close cooperation with the information technology department - Understanding the hospital dynamics and hospital project development process specifications in a timely manner and synchronizing the standard operating procedure of the center project development within the company |
|  | **Deployment and implementation** | |
|  |  | - Tracking and maintenance of the data interface - Quality control of data after docking and synchronization |
|  | **Project promotion** | |
|  |  | - Coordinate the internal project configuration members of the company and configure the project based on the project plan and electronic case report form collection interface (database creation) - If the project needs to extract and manage the source data, coordinate the company’s internal natural language processing engineer training model - If the project needs to customize personalized functions, coordinate with the product manager to design and develop functional pages |
| **Hospital research management department** | | |
|  | **Project approval** | |
|  |  | - At the time of project approval, the project team can submit the application for data synchronization in the institution, stating the scope of data synchronization, the synchronization frequency, the responsible person, and the implementation start and end dates - The information technology department is included in the application for the in-hospital data synchronization batch - Data collection and governance methods are reflected in the research plan and research indicators are generated by synchronizing the information system data in the hospital and extracting governance through natural language processing - Establish a quality control mechanism and quality control plan for synchronizing hospital data as research source data |
|  | **Ethics** | |
|  |  | - Establish clear instructions for the project data collection and management methods, for example, regarding whether to agree to the project collection and synchronization of the hospital information system data as a supplementary means of data collection - Establish a processing mechanism for changes in the scope, method, operator, and so on, of data synchronization during the subsequent implementation of the project |
|  | **Launch** | |
|  |  | - The organization secretary grants data synchronization authority for the members of the project team - Evaluate the quality of the source data, propose the place where the writing is not standardized, and correct it later - Changes in the scope of data synchronization, an increase in the number of data synchronization operators and other changes during project implementation must be reported to the institution or ethics office for filing in a timely manner |
| **Researcher or project team** | | |
|  | **Preparation** | |
|  |  | - Analyze and determine the source, method, scope, system, and project members involved in source data collection - The project is configured and tested in the ESR - Hospital project application and ethics meeting |
|  | **Collection** | |
|  |  | - Researchers regularly synchronize the source data - The CRC^b^ data check the study data - Investigator data query cleaning - CRC assistance with subject management and follow up outside the hospital |
|  | **Quality control** | |
|  |  | - Accept the regular quality control of the project by the quality controller of the good clinical practice - Accept the sponsor’s verification of the project - Cooperate with the organization and the sponsor to establish the quality control plan of the source data synchronization of the project and follow the corresponding standard operating procedure - On the basis of project implementation, adjust and supplement the standard operating procedure and plan to reduce risks in a timely manner |

^a^ESR: eSource record.

^b^CRC: clinical research coordinator.
